# 259. Comparison of the causes of death in patients with delta variant versus omicron variant infections

**DOI:** 10.1093/ofid/ofac492.337

**Published:** 2022-12-15

**Authors:** A Reum Kim, Jiyoung Lee, Somi Park, Sung Woon Kang, Yun Woo Lee, So Yun Lim, Euijin Jang, SeongMan Bae, Jiwon Jung, Min Jae Kim, Yong Pil Chong, Sang-Oh Lee, Yang Soo Kim, Sung-Han Kim

**Affiliations:** Department of Infectious Disease, Asan Medical Center, University of Ulsan College of Medicine, Seoul, Seoul-t'ukpyolsi, Republic of Korea; Asan medical center, Seoul, Seoul-t'ukpyolsi, Republic of Korea; Asan medical center, Seoul, Seoul-t'ukpyolsi, Republic of Korea; Asan medical center, Seoul, Seoul-t'ukpyolsi, Republic of Korea; Asan Medical Center, Seoul, Seoul-t'ukpyolsi, Republic of Korea; Asan medical center, Seoul, Seoul-t'ukpyolsi, Republic of Korea; Asan medical center, Seoul, Seoul-t'ukpyolsi, Republic of Korea; Asan medical center, Seoul, Seoul-t'ukpyolsi, Republic of Korea; Asan Medical Center, Seoul, Seoul-t'ukpyolsi, Republic of Korea; Asan Medical Center, Seoul, Seoul-t'ukpyolsi, Republic of Korea; Asan Medical Center, Seoul, Seoul-t'ukpyolsi, Republic of Korea; Asan Medical Center, Seoul, Seoul-t'ukpyolsi, Republic of Korea; Asan Medical Center, Seoul, Seoul-t'ukpyolsi, Republic of Korea; Asan medical center, Seoul, Seoul-t'ukpyolsi, Republic of Korea

## Abstract

**Background:**

Severe acute respiratory syndrome-coronavirus-2 (SARS-CoV-2) variant strain B.1.1.529 (omicron) has been less virulent than SARS-CoV-2 B.1.617.2 variant (delta), but there are limited data on the comparison of the cause of death between delta variant and omicron variant infections. We thus compared the causes of death in COVID-19 patients with the delta variant and omicron variant.

**Methods:**

We retrospectively reviewed the medical records of adult patients with COVID-19 who were admitted at Asan Medical Center, Seoul, South Korea, between July 2021 and March 2022. We divided into delta-variant dominant period (from July 2021 to December 2021) and omicron-dominant period (from February 2022 to March 2022) with the exclusion of January 2022 because this period was overlapping of delta and omicron variant. The causes of death were classified into COVID-19-associated pneumonia, other causes, and indeterminate cause.

**Results:**

A total of 654 patients with COVID-19 were admitted and 42 (6.4%) died during the omicron dominant period (between February and March 2022), while a total of 366 patients with COVID-19 were hospitalized and 42 (11.5%) died during the delta dominant period (between July and December 2021). The primary cause of death was COVID-19-associated pneumonia in 64% (27/42) during the omicron era whereas that was COVID-19-associated pneumonia in 88% (37/42) during the delta era (p value=0.01) (Table 1).

**Figure:**
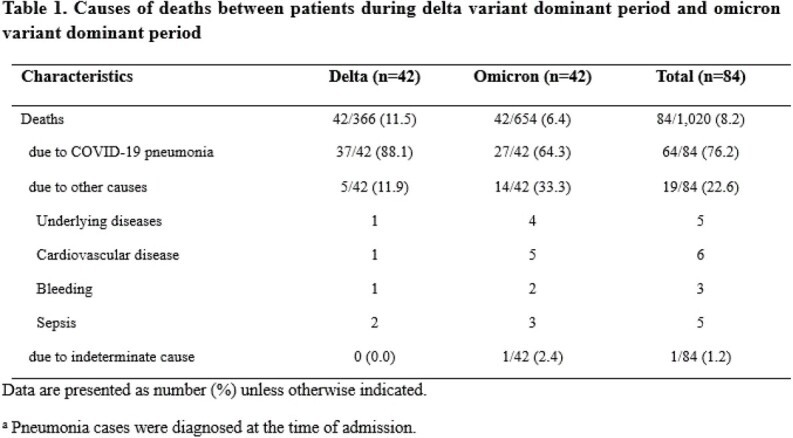

**Conclusion:**

We found that about two thirds of patients with omicron variant infection died due to COVID-19, while the majority of patients with delta variant infection died due to COVID-19.

**Disclosures:**

**All Authors**: No reported disclosures.

